# Arsenic-Associated Oxidative Stress, Inflammation, and Immune Disruption in Human Placenta and Cord Blood

**DOI:** 10.1289/ehp.1002086

**Published:** 2010-10-12

**Authors:** Sultan Ahmed, Sultana Mahabbat-e Khoda, Rokeya Sultana Rekha, Renee M. Gardner, Syeda Shegufta Ameer, Sophie Moore, Eva-Charlotte Ekström, Marie Vahter, Rubhana Raqib

**Affiliations:** 1 International Centre for Diarrhoeal Disease Research, Bangladesh, Dhaka, Bangladesh; 2 Institute of Environmental Medicine, Karolinska Institutet, Stockholm, Sweden; 3 Medical Research Council International Nutrition Group, London School of Hygiene and Tropical Medicine, London, United Kingdom; 4 International Maternal and Child Health, Department of Women’s and Children’s Health, Uppsala University Hospital, Uppsala, Sweden

**Keywords:** arsenic, cytokines, 8-oxoguanine, leptin, oxidative stress, placenta

## Abstract

**Background:**

Arsenic (As) exposure during pregnancy induces oxidative stress and increases the risk of fetal loss and low birth weight.

**Objectives:**

In this study we aimed to elucidate the effects of As exposure on immune markers in the placenta and cord blood, and the involvement of oxidative stress.

**Methods:**

Pregnant women were enrolled around gestational week (GW) 8 in our longitudinal, population-based, mother–child cohort in Matlab, an area in rural Bangladesh with large variations in As concentrations in well water. Women (*n* = 130) delivering at local clinics were included in the present study. We collected maternal urine twice during pregnancy (GW8 and GW30) for measurements of As, and placenta and cord blood at delivery for assessment of immune and inflammatory markers. Placental markers were measured by immunohistochemistry, and cord blood cytokines by multiplex cytokine assay.

**Results:**

In multivariable adjusted models, maternal urinary As (U-As) exposure both at GW8 and at GW30 was significantly positively associated with placental markers of 8-oxoguanine (8-oxoG) and interleukin-1β (IL-1β); U-As at GW8, with tumor necrosis factor-α (TNFα) and interferon-γ (IFNγ); and U-As at GW30, with leptin; U-As at GW8 was inversely associated with CD3^+^ T cells in the placenta. Cord blood cytokines (IL-1β, IL-8, IFNγ, TNFα) showed a U-shaped association with U-As at GW30. Placental 8-oxoG was significantly positively associated with placental proinflammatory cytokines. Multivariable adjusted analyses suggested that enhanced placental cytokine expression (TNFα and IFNγ) was primarily influenced by oxidative stress, whereas leptin expression appeared to be mostly mediated by As, and IL-1β appeared to be influenced by both oxidative stress and As.

**Conclusion:**

As exposure during pregnancy appeared to enhance placental inflammatory responses (in part by increasing oxidative stress), reduce placental T cells, and alter cord blood cytokines. These findings suggest that effects of As on immune function may contribute to impaired fetal and infant health.

Chronic exposure to inorganic arsenic (iAs) through elevated concentrations in drinking water is a major environmental health hazard throughout the world, particularly in Bangladesh and India, resulting in increased risk of cancer and numerous noncancer effects (International Agency for Research on Cancer 2004). iAs is readily transferred to the fetus (Concha et al. 1998), and recent studies have shown increased rates of fetal loss, preterm births, and neonatal mortality (Ahmad et al. 2001; Milton et al. 2005; Rahman et al. 2007), as well as decreased birth weight (Hopenhayn et al. 2003; Rahman et al. 2009), at fairly low exposure levels. The mechanisms of arsenic (As)-induced developmental toxicity are, however, poorly understood (Vahter 2009).

One of the mechanisms of As toxicity is generation of reactive oxygen species, possibly in combination with impaired antioxidative defense (Vahter 2009; Wu et al. 2001). Our ongoing, prospective, mother–child cohort study in rural Bangladesh has shown that women in early gestation have As-related increases in urinary 8-oxo-7, 8-dihydro-2′-deoxyguanosine (8-OHdG), a product of oxidative DNA damage that is used as a marker of oxidative stress (Engstrom et al. 2010). Oxidative stress in the placenta may trigger secretion of proinflammatory factors and has been associated with preeclampsia (Cindrova-Davies et al. 2007; Myatt 2006).

As has also been shown to induce immunosuppression in epidemiological and experimental studies. Our ongoing studies in Bangladesh showed that exposure to As during pregnancy was associated with decreased thymic size in the children and increased infectious diseases in both mothers and children, indicating As-related immunosuppression (Moore et al. 2009; Rahman et al. 2010; Raqib et al. 2009). *In vitro* and *in vivo* studies have shown reduced lymphocyte proliferation, reduced CD4^+^ cell counts, CD4^+^:CD8^+^ cell ratios, and reduced T-regulatory cells in exposed adults and children (Biswas et al. 2008; Hernandez-Castro et al. 2009; Soto-Pena et al. 2006). Experimental studies in mice have also shown dose-dependent increases in apoptosis of thymocytes and splenocytes (Stepnik et al. 2005) and decreased bacterial clearance from the blood and spleen after chronic As exposure (Bishayi and Sengupta 2003). In this study, we aimed to increase our understanding of the impact of As exposure during pregnancy on immune function in terms of immune and inflammatory markers in the placenta and cord blood in association with oxidative stress.

## Materials and Methods

### Study area and subjects

The study area, Matlab, is located 53 km southeast of Dhaka, Bangladesh. The International Centre for Diarrhoeal Disease Research, Bangladesh, runs a health and demographic surveillance system (HDSS) in Matlab, as well as a central hospital and four connected subcenters that provide health care to the resident population in the areas. About 70% of the tube wells in Matlab exceed the World Health Organization guideline level of As in drinking water of 10 μg/L, and 50% exceed the national standard of 50 μg/L (Rahman et al. 2006).

This study is nested in a large, randomized, population-based food and multimicronutrient supplementation trial [Maternal and Infant Nutrition Interventions, Matlab (MINIMat) trial; ISRCTN16581394], which evaluated nutritional and environmental impacts on pregnancy outcomes and child health (Moore et al. 2009; Raqib et al. 2009; Tofail et al. 2008). Pregnancy was identified by urine test in women (*n* = 3,600) who reported missing a menstrual period at monthly home visits by HDSS workers. Once pregnancy was identified, usually during gestational weeks (GW) 6–10 (mean, GW8), women were advised to visit a health facility in their area for confirmation of pregnancy by ultrasound and for antenatal care. Women were then invited to enroll into the MINIMat trial and were requested to donate urine samples at enrollment (GW8) and at GW30 and cord blood and placenta at delivery. In the MINIMat trial, women were randomized to receive one of three different combinations of micronutrient supplements, daily from GW14 up to birth: *a*) 30 mg iron plus 400 μg folic acid, *b*) 60 mg iron plus 400 μg folic acid, and *c*) the UNICEF preparation of 15 different micronutrients including 30 mg iron and 400 μg folic acid. Participants gave written, informed consent, and the study was approved by the ethical review committee of International Centre for Diarrhoeal Disease Research, Bangladesh. The women could not be informed about their As status during their pregnancy because these analyses were done later.

Field research assistants collected morbidity information, based on a set of structured questionnaires, from pregnant women during the scheduled monthly home visits. The questionnaires included four specific morbidity questions concerning diarrhea/dysentery, respiratory illness (in terms of cold, cough, or difficult breathing), and urinary tract infections (in terms of pain/burning/difficulty during urination) with or without concomitant fever, including the duration of the morbidity symptoms (days of illness). Socioeconomic status (SES) was based on family assets using data from the HDSS database (Raqib et al. 2009; Tofail et al. 2008).

The present cohort consists of a convenience sample of 130 women delivering singleton infants at the central Matlab hospital or any of the four connected subcenters during early daytime (0500 to 1430 hours). Home deliveries and later deliveries could not be included because of logistic difficulties of processing and transferring samples to the Dhaka laboratory. Birth weight was measured within 72 hr of delivery using electronic scales (Seca pediatric scales; Seca GmbH, Hamburg, Germany) with precision of 10 g. The infant’s length was measured using a validated, locally manufactured wooden length board, with precision of 0.1 cm.

### As exposure

The concentration of metabolites of iAs in urine was used as a biomarker of exposure because it reflects the ingested dose of iAs from all sources (Vahter et al. 2006). Although the half-life of iAs in the body is relatively short, the daily exposure through water and food results in fairly stable steady-state concentrations in urine. In the larger MINIMat cohort, we observed that maternal urinary As (U-As) is tightly correlated with blood As levels (*r*^2^ = 0.83, *p* < 0.001; unpublished data). As in hair and nail samples, used sometimes as biomarkers of As exposure over several months, were not considered in this highly exposed population because of risk of external exposure contamination, for example, bathing and washing in contaminated water. Because As easily crosses the placenta (Concha et al. 1998), the concentration in maternal urine during pregnancy was used as a proxy marker of fetal exposure.

Urine was collected into trace element–free plastic cups, transferred to 24‐mL polyethylene bottles, and stored at −70°C in the laboratory. The mothers’ U-As, defined as the sum of iAs and iAs metabolites in urine (iAs + methylarsonic acid + dimethylarsinic acid), was determined using hydride generation atomic absorption spectrophotometry (Vahter et al. 2006). The limit of detection (LOD) was 1.3 ± 0.27 μg/L. To compensate for variation in dilution, urine samples were adjusted for specific gravity measured by a digital refractometer (RD712 Clinical Refractometer; EUROMEX, Arnhem, Holland). Adjustment by specific gravity has been shown to be less affected by body size, SES, and As exposure than the more commonly used creatinine adjustment (Nermell et al. 2008).

### Processing of placental tissue for immunostaining

Placental tissue was collected from mothers immediately after delivery at the clinic. A 2 × 2-inch piece from the fetal portion of the placenta was cut and immersed in 10% buffered formalin (pH 7.0) kept at room temperature (RT). Smaller pieces were cut from the placental specimen and divided into two sets. One set was fixed, embedded in paraffin, sectioned by Microtome (Leica, Nussloch, Germany) at 3‐mm thickness, mounted on glass slides treated with Vectabond reagent (Vector Laboratories, Burlingame, CA, USA), dried overnight at 37°C, and kept at RT before use for immunostaining. The second set of tissues was immersed in 15% sucrose buffer overnight and, after removal of excess fluid, was snap-frozen in liquid nitrogen to retain the antigenic properties.

### Immunohistochemical detection of cell surface markers and proinflammatory cytokines

Paraffin-embedded tissue sections were deparaffinized and microwave-treated in target retrieval solution (pH 6.0–6.2) for antigen retrieval. After blocking endogenous peroxidase activity and nonspecific staining, the slides were incubated sequentially with primary antibodies (overnight at RT), antibody conjugate, and avidin-biotin complex at 37°C for 30 min, each with intermittent washing. Positive staining was developed with substrate 3,3′-diaminobenzidine (Sigma-Aldrich, St. Louis, MO, USA). Sections were counterstained with hematoxylin, dehydrated, and mounted. Frozen tissue blocks were sectioned with a cryomicrotome (Leica) at 4–5 μm thickness. Staining for CD64 and cytokines was performed in the cryostat sections as described previously (Raqib et al. 1995). Primary antibodies used were anti-human: CD3 (pan T cell), CD8 (cytotoxic/suppressor T cells), CD64 (FcgRI, monocytes/macrophages), CD68 (phagocytes), myeloperoxidase (MPO; neutrophils), interleukin-1β (IL-1β), and iterferon-γ (IFNγ) (all above from DakoCytomation, Glostrup, Denmark); leptin ob (Y-20) (Santa Cruz Biotechnology, Inc., Santa Cruz, CA, USA); tumor necrosis factor-α (TNFα) (BD Biosciences, Sparks, MD, USA); IL-6 (BD Pharmingen, San Diego, CA, USA); IL-10 (Mabtech, Cincinnati, OH, USA); and 8-oxoguanine (8-oxoG) (Chemicon International, Temecula, CA, USA).

### Image analysis of immunostaining

Immunohistochemical staining of specific markers in the placental specimens was analyzed with a microscope (Olympus BX51; Olympus Optical Co. Ltd., Tokyo, Japan) connected to a CCD camera (DCF295; Leica Microsystems CMS GmbH, Wetzlar, Germany) and the image analysis system Leica Qwin Runner (version 3). Special software (Tissue-includer), written in the high-level language QUIPS, was used to examine each video microscopic digital image. The color and morphology of the hematoxylin counterstained cells were set as a standard for positive and negative cells, and the positive staining of markers in placental tissue sections was defined by Leica software (Sarker et al. 2009). The acquired video microscopic image was divided into 512 × 512 pixels, and each pixel was expressed in square micrometers (area) after calibration with the current magnification. For each placental tissue section, all fields (average, 400 ± 25 fields) were investigated at 400× magnification, and the average of all fields was used for quantification of immunostaining in each tissue section. The results were expressed as {(total positively stained area) × (total mean intensity [1–256 levels per pixel] of the positive area)} / total cell area, in units referred to as acquired computerized image analysis (ACIA) scores [see Supplemental Material, “Computerized Image Analysis for Detection of Immunostaining,” and Supplemental Material, Figure 1 (doi:10.1289/ehp.1002086)].

For T-cell (CD3^+^, CD8^+^) staining, the computerized image analysis technique could not be applied because the numbers of cells in the total tissue section were very small, so ACIA scores were very low. T-cell phenotypes in the placental sections were assessed manually using an ocular micrometer fitted into the eyepiece of the microscope (Olympus Optical Co. Ltd.), and data were calculated as number of cells per 100‐μm^2^ field area. Two trained individuals counted 400 fields separately, and the average was used.

### Cytokines assay in cord blood plasma

Cord blood specimens were collected in heparin-coated sterile vials (Becton Dickinson, Rutherford, NJ, USA) at the subcenter health clinics at delivery and were transported on ice to the laboratory. Plasma was separated and stored at −70°C until analyzed by Bio-Plex 200 reader (BioRad Laboratories, Hercules, CA, USA). Cytokine levels in plasma were determined using Bio-Plex Pro Human Cytokine 18-Plex Panel [IL-1β, IL-2, IL-4, IL-5, Il-6, IL-7, IL-8, IL-10, IL-12 (p70), IL-13, IL-17, granulocyte colony-stimulating factor, granulocyte-macrophage colony-stimulating factor, IFNγ, monocyte chemotactic protein-1, macrophage inflammatory protein-1β, TNFα, and human interferon-inducible protein-10] according to the manufacturer’s instructions. The LOD was approximately 10 pg/mL for these cytokines. For cytokine measurements with values below LOD, the LOD divided by square root of 2 was used.

### Statistical analysis

Statistical analyses were performed using the software PASW 18.0 (SPSS Inc., Chicago, IL, USA). Data distribution pattern (normality) and homogeneity of variances were checked. Concentrations and ACIA score data were log transformed if not normally distributed. Covariates [maternal age, morbidity, body mass index (BMI), gestational age, parity, SES, tobacco chewing (TC), birth weight, supplementation group, sex of the children] were evaluated for significant association with exposure and outcomes. Spearman’s correlation was used to evaluate univariate associations between covariates and U-As (GW8 and GW30) and immune and inflammatory markers in cord blood and placenta. Significantly correlated variables were further analyzed in multivariable adjusted regression models controlling for potential confounders. Potential confounders were identified based on correlations (*p* < 0.15) with the exposure (As) and outcome and were included in final models when they changed the effect estimate for U-As on the outcome by 5% or more. Mothers’ age, SES, and TC were included in the final model. Cord blood cytokines were analyzed according to quartiles of As exposure (*n* = 130; i.e., 32–33 in each quartile). Analysis of covariance (ANCOVA) was done to analyze the association of As and cord blood cytokines by adjusting for the same confounding factors as above if the association was significant in one-way analysis of variance (ANOVA). *p*-Values < 0.05 were considered significant.

## Results

### Demographic data and As exposure

The average age of the pregnant women was 25.5 years, and the average gestation age at delivery was 39.5 weeks, with small variations (Table 1). Of the 130 births, 68 were male and 62 were female. Twenty infants (8 males and 12 females) were of low birth weight (< 2,500 g). The range of parity was 0 to 7. All women were nonsmokers; however, 22% of the women reported TC. The “chewing tobacco” consists of dried tobacco leaves locally known as *zarda* (sweetened tobacco leaves) or *gul* (dried and powdered tobacco). *Zarda* is taken in a mixture of sliced areca nut, lime, and a leaf of the piper betel plant, whereas *gul* is placed between the gums to be slowly sucked (Lindberg et al. 2010). The median U-As concentrations at GW8 and GW30 were similar at 66 and 60 μg/L, respectively. However, we observed considerable interindividual variation in As exposure. Although U-As in GW8 and GW30 were significantly correlated (*r* = 0.599, *p* < 0.001), 15 women showed decreasing concentrations by > 50 μg/L, whereas 20 women showed increasing concentrations by > 50 μg/L from early to late gestation. Therefore, we evaluated the outcomes by maternal U-As in early and late gestation separately.

### Maternal As exposure and placental immune markers

Localization of the various markers in the placenta was generally same for all subjects. We generally found increased expression of the various cytokines and reduced cell counts/expression at high exposure levels [median U-As > 60 μg/L at GW30; see Supplemental Material, Figure 2 and “Localization of immune cells and inflammatory markers in the placenta” (doi:10.1289/ehp.1002086)].

### Maternal As exposure and placental immune cells

**Figure 1:**
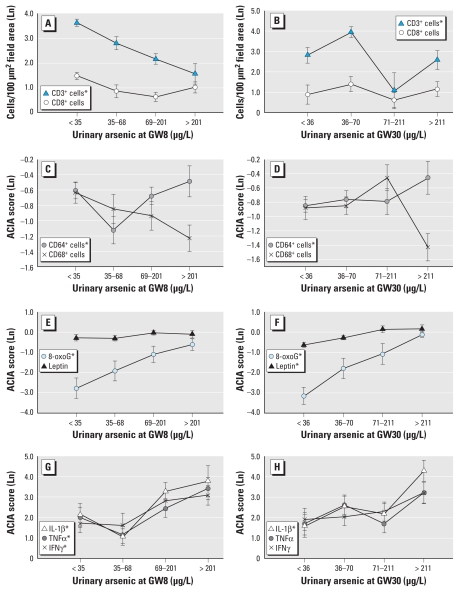
(*A* and *B*) Association between U-As and frequencies of placental CD3^+^ and CD8^+^ cells, using quartiles of U-As concentrations at GW8 (*A; n* = 130, 32–33 in each quartile; median U-As, 26, 46, 115, and 341 μg/L, respectively) and GW30 (*B; n* = 130, 32–33 in each quartile; median U-As, 27, 48, 121, and 335 μg/L, respectively). (*C* and *D*) Association between U-As and expression of CD64^+^ and CD68^+^ cells, using quartiles of U-As exposure at GW8 (*C*) and GW30 (*D*). (*E* and *F*) Association between U-As and 8-oxoG and leptin expression, using quartiles of U-As exposure at GW8 (*E*) and GW30 (*F*). (*G* and *H*) Association between U-As and TNFα, IFNγ, and IL-1β expression, using quartiles of U-As concentrations at GW8 (*G*) and GW30 (*H*). Data are means ± SE. Ln, natural log. *p < 0.05 for U-As modeled as a continuous variable in multivariable-adjusted linear regression analysis (Table 2).

Evaluation of associations between As exposure during pregnancy (U-As at GW8 and GW30) and the phenotypic expression of immune cells in the placenta (CD3^+^, CD8^+^, CD64^+^, CD68^+^, MPO) by regression analysis showed a significant negative association between CD3^+^ cell frequency in the placenta and U-As at GW8 but not at GW30 (Table 2, Figure 1A,B). The association remained significant after adjusting for age, SES, and TC (Table 2). Placental CD8^+^ cells decreased with increasing U-As at GW8, but the trend was not significant (Table 2, Figure 1A). The CD64^+^ cells were significantly negatively associated with U-As at GW8 but not at GW30 (Table 2, Figure 1C,D). We found no significant association between maternal U-As at GW8 or GW30 and placental expression of CD68 (phagocytes) and MPO [neutrophils; see Supplemental Material, Table 1 (doi:10.1289/ehp.1002086)]. We found no significant effect of micronutrient supplementation on placental or cord blood outcome variables and no difference in As exposure among supplementation groups. We found no significant association between the sex of the infant and placental and cord blood immune parameters or As exposure.

### Maternal As exposure and cytokines and oxidative damage in placenta

In regression analyses (Table 2), we found significant positive associations between U-As at both GW8 and GW30 and the placental expression of 8-oxoG (Figure 1E,F), IL-1β, TNFα, and IFNγ (Figure 1G,H). Placental expression of leptin was significantly associated with U-As at GW30 but not at GW8 (Figure 1E,F). We found no significant associations between IL-6 or IL-10 and U-As [see Supplemental Material, Table 1 (doi:10.1289/ehp.1002086)]. In multivariable adjusted linear regressions analysis (adjusting for age, SES, and TC), U-As at both GW8 and GW30 was significantly associated with placental 8-oxoG and IL-1β; U-As at GW8, with TNFα and IFNγ; and U-As at GW30, with leptin (Table 2).

Placental 8-oxoG was significantly associated with several placental cytokines (leptin, IL-1β, TNFα, and IFNγ) (Table 3). To investigate whether the estimated effects of As on placental cytokines may have been mediated via oxidative stress, we performed multivariable adjusted linear regression with placental cytokines (leptin, IL-1β, TNFα, and IFNγ) as dependent variables and U-As as independent variable, controlling for 8-oxoG and potential confounders (Table 3). All the associations between placental cytokines and U-As at GW8 did not remain significant after adjustment for 8-oxoG. The association between U-As at GW30 and placental leptin decreased slightly but remained significant after adjustment for 8-oxoG. The association between U-As at GW30 and IL-1β also decreased and was no longer significant after adjusting for 8-oxoG. Coefficients for the association of U-As at GW30 with placental IFNγ and TNFα were decreased markedly after adjustment for 8-oxoG (in addition to age, SES, and TC), but estimates for associations of 8-oxoG with placental IFNγ and TNFα remained positive and significant when adjusted for U-As at GW30.

In multivariable adjusted linear regression, placental expression of IL-6 and IL-10 was significantly associated with the number of days of maternal urinary tract infection during pregnancy (β-coefficient = 0.45, *p* = 0.044) and acute respiratory infection (β-coefficient = 0.50, *p* = 0.017), respectively, after adjustment for U-As and other potential confounders. We observed no significant associations between mothers’ other morbidities (fever and diarrhea) and other markers in the placenta and cord blood.

### Maternal As exposure and cord blood cytokines

**Figure 2:**
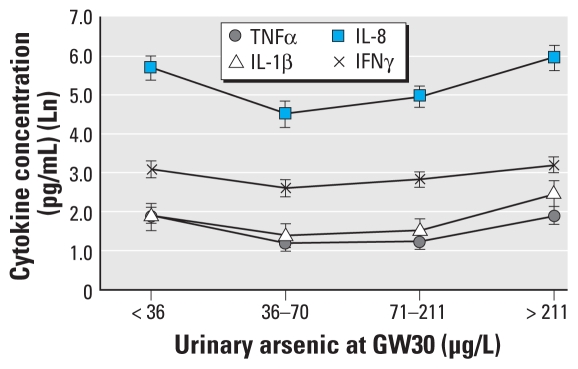
Association of As exposure at GW30 with cord blood cytokines (TNFα, IL-1β, IL-8, and IFNγ). Concentrations of cord blood cytokines in different quartiles of U-As concentrations at GW30 (as in Figure 1H) are expressed as mean ± SE. Ln, natural log.

For all cytokines with the exception of IL-5, > 90% of the plasma samples were above the LOD of the multiplex cytokine assays (Bio-Plex) (data not shown). In the case of IL-5, 50% of the samples were below LOD. Cord blood proinflammatory cytokines TNFα, IL-8, IL-1β, and to some extent IFNγ showed U-shaped curves by quartiles of U-As at GW30 (Figure 2) but not at GW8 (data not shown). In ANOVA post hoc test (least significant difference) using quartiles of U-As at GW30, we observed a significant difference between second and the fourth quartiles for log-transformed IL-8 [means and 95% confidence intervals (CIs) in the second and fourth quartiles were 4.51 (3.83–5.18) pg/mL and 5.96 (5.30–6.61) pg/mL, respectively] and log-transformed TNFα [means (95% CIs) in the second and fourth quartiles were 1.20 (0.77–1.63) pg/mL and 1.90 (1.44–2.34) pg/mL, respectively]. IL-1β also showed an increase at higher U-As levels; however, the difference was not significant (*p* = 0.09). In the ANCOVA analyses, after adjusting for age, SES, and TC, we observed significant differences only for IL-8 (*p* = 0.012) but not for TNFα. We observed no significant associations between maternal As exposure and the rest of the 14 cord blood cytokines evaluated (data not shown).

## Discussion

The results of this study suggest that maternal exposure to As during pregnancy increases oxidative stress and inflammation in the placenta. The measured placental proinflammatory cytokines (IL-1β, TNFα, and IFNγ) were mainly influenced by oxidative stress, part of which might have been mediated by As. To our knowledge, this is the first report of As-associated leptin expression in the placenta, apparently independent of oxidative stress. Maternal exposure in early pregnancy was also associated with decreased pan T cells (CD3^+^ cells) in the placenta but apparently not with other cell types studied. We also report preliminary evidence that maternal As exposure showed a U-shaped association with proinflammatory cytokine concentrations in cord blood.

Reactive oxygen species produced by As are known to induce a wide range of lesions, including DNA lesions (Xu et al. 2008). We have previously reported As-related increase in 8-OHdG in urine in the MINIMat cohort (Engstrom et al. 2010). Earlier studies have assessed oxidative stress in tissues by immunostaining of 8-oxoG, a marker of nuclear DNA damage (Nakae et al. 2005). In this study, immunohistochemical localization of 8-oxoG in the placenta, particularly in the cytotrophoblasts, syncytiotrophoblasts, and capillary endothelium, showed a strong association with U-As. Oxidative stress is known to induce the release of proinflammatory factors, including cytokines, in the placenta that act in autocrine, paracrine, and juxtracrine manners for diverse functions (Cindrova-Davies et al. 2007). Our models showed that the significant relationships between maternal U-As and placental proinflammatory cytokines were no longer significant after adjustment for 8-oxoG, suggesting that the cytokine production was mainly influenced by oxidative stress, only part of which appeared to be mediated by As. However, both As and oxidative stress seemed to play an important role in placental proinflammatory IL-1β production.

We observed U-shaped concentration–response curves for cord blood proinflammatory cytokines IL-8 and TNFα; however, we observed no relationship between maternal As exposure and anti-inflammatory or regulatory cytokines. Cord blood proinflammatory cytokines IL-6 and IL-8 are used as a marker of early-onset infection in premature infants (Krueger et al. 2001). Some caution must be used when comparing the cytokines measured in cord blood with those measured in placenta, because different methods are used. There is a lack of studies of cord blood cytokine levels in healthy neonates (Lyon et al. 2009), so it is difficult to interpret the As-induced changes in cord blood cytokines in the present study.

Immune tolerance at the maternal–fetal interface is established with decreased cell-mediated immunity and increased humoral immunity. A diverse array of both proinflammatory and anti-inflammatory cytokines are released by the trophoblasts with an overall shift toward anti-inflammatory mediators, thereby promoting maternal tolerance (Hunt et al. 2010). In the present study, high As exposure was associated with a shift toward proinflammatory cytokines (IL‐1β, TNFα, IFNγ, and IL-8) in both placenta and cord blood. Proinflammatory cytokines (TNFα, IFNγ) were found to induce apoptosis of placental trophoblasts *in vitro* (Yui et al. 1994). In addition, we found that morbidity during pregnancy was associated with increased placental expression of IL-6 and IL-10, indicating that placental cytokine expression also changes in response to clinically relevant events. Maternal circulating proinflammatory cytokines (IL-1β, TNFα) induced by infection or inflammation have previously been associated with preeclampsia (Rinehart et al. 1999). Elevated levels of cord blood proinflammatory cytokines (IL‐1β, TNFα, and IL-6) have also been associated with preterm birth, one of the most common causes of neonatal morbidity and mortality (Lyon et al. 2009), as well as chorioamnionitis, brain white matter damage, and chronic lung disease in preterm infants (An et al. 2004; Kaukola et al. 2006).

*In vitro* As exposure at higher levels induces apoptosis in immune cells (de la Fuente et al. 2002). *In vitro* and *in vivo* investigations showed significant inverse correlation between chronic As exposure and the number and function of T-regulatory cells in adults chronically exposed to As (Hernandez-Castro et al. 2009). We report that maternal As exposure was associated with a decrease in the number of pan T cells in the placenta in a dose-dependent manner in the presence of elevated levels of inflammatory cytokines (Table 2, Figure 1A). In particular, the CD4^+^CD25^+^ regulatory T cells (Treg cells) and regulatory natural killer cells play important roles in the maintenance of immune tolerance in the maternal–fetal interface. During preeclampsia, Treg cells are down-regulated in the placenta compared with normal pregnancy (Sasaki et al. 2007). It is possible that apoptotic deletion of CD4^+^ Treg cells among the total CD3^+^ cells (given no significant changes in the CD8^+^ cell numbers) leads to an imbalance in the placental immune tolerance. One limitation of this study is that we did not measure CD4^+^ cells in placenta. We noted no significant association between CD3 or other immune cell markers and 8-oxoG expression, suggesting that the suppressive effect of As on T-lymphocyte numbers is probably mediated by pathways other than oxidative stress.

As exposure in late gestation was significantly associated with increased placental leptin expression, starting at fairly low As exposure levels (consumption of < 50 μg/L in drinking water). Leptin is tightly regulated and involved in key processes in fetal development, including transfer of nutrients from placenta to the developing fetus (Jansson et al. 2003; White et al. 2006). Placental leptin production is increased during hypoxia (Spranger et al. 2008) and in various obstetrical conditions such as diabetes, preeclampsia, and intrauterine growth retardation (Iwagaki et al. 2004; Mise et al. 1998). Further studies are necessary to understand the potential health effects of As-induced leptin expression in the placenta.

## Conclusion

As exposure during pregnancy was associated with oxidative stress and increased inflammatory cytokines and leptin in the placenta. Furthermore, As exposure in early pregnancy was associated with reduced T-cell numbers in the placenta, indicating a disruption of immune balance. It is possible that such changes may influence fetal programming and increase susceptibility to infectious diseases postnatally (Jansson and Powell 2007). Indeed, we have observed an As-associated increase in infant morbidity in infectious diseases (Rahman et al. 2010; Raqib et al. 2009). Therefore, effects of prenatal As exposure on immune function may also have consequences for susceptibility to various diseases later in life.

## Figures and Tables

**Table 1:** Demographic data of the study group of 130 mother–child pairs.

Characteristic	Value
Pregnant women	

Age of women at enrollment (years)	
Mean ± SD	25.5 ± 5.9
Median (10th–90th percentiles)	24 (17–36)
BMI at GW8 (kg/m^2^)	
Mean ± SD	20.6 ± 2.9
Median (10th–90th percentiles)	19.86 (17.08–24.60)
BMI at GW30 (kg/m^2^)	
Mean ± SD	23.0 ± 2.6
Median (10th–90th percentiles)	22.37 (19.83–26.52)
Gestational age (weeks)	
Mean ± SD	39.5 ± 1.5
Median (10th–90th percentiles)	39 (37–41)
Supplementation group^a^	
1	43 (33%)
2	42 (32%)
3	45 (35%)
Parity	
0	55 (42%)
1–2	54 (42%)
3–7	21 (16%)
Socioeconomic status	
Low	38 (29%)
Middle	26 (20%)
High	66 (51%)
U-As at GW8 (μg/L)^b^	
Median (10th–90th percentiles)	66 (23–343)
Mean ± SD	136 ± 167
U-As at GW30 (μg/L)^b^	
Median (10th–90th percentiles)	60 (20–374)
Mean ± SD	143 ± 164
Tobacco chewing	
Yes	29 (22%)
No	101 (78%)

Children	

Birth weight (g)	
Mean ± SD	2,786 ± 385
Median (10th–90th percentiles)	3,794 (2,353–3,300)
Low birth weight (< 2,500 g)	20 (16.0%)
Birth length (cm)	
Mean ± SD	47.0 ± 1.9
Median (10th–90th percentiles)	48 (45–50)
Girls	62 (48.0%)

Values shown are *n* (%) unless otherwise noted.

a Supplementation groups: 1, 30 mg iron plus 400 μg folic acid; 2, 60 mg iron plus 400 μg folic acid; 3, the UNICEF preparation of 15 different micronutrients including 30 mg iron and 400 μg folic acid.

b Adjusted to average specific gravity of 1.012 g/mL.

**Table 2:** Multivariable adjusted analysis of associations of maternal U-As (μg/L) in early and late pregnancy with placental effect markers, for CD3^+^ and CD8^+^ cells (cells/100 μm^2^ field area) and for other cells and cytokines (ACIA score).

	Independent variable			
	Unadjusted		Adjusted^a^	
Dependent variable	U-As at GW8	U-As at GW30	U-As at GW8	U-As at GW30
CD3				

β-Coefficient	−0.64	−0.44	−0.66	−0.46
95% CI	−1.07 to −0.20	−0.90 to 0.03	−1.19 to −0.12	−1.04 to 0.12
*p*-Value	0.004	0.066	0.017	0.11

CD8				

β-Coefficient	−0.20	−0.08	−0.20	0.07
95% CI	−0.55 to 0.15	−0.45 to 0.30	−0.66 to 0.25	−0.56 to 0.41
*p*-Value	0.26	0.68	0.37	0.76

CD64				

β-Coefficient	−0.29	−0.18	−0.28	−0.18
95% CI	−0.57 to −0.01	−0.49 to 0.12	−0.68 to 0.10	−0.61 to 0.23
*p*-Value	0.04	0.24	0.14	0.37

8-oxoG				

β-Coefficient	0.78	1.08	0.79	1.07
95% CI	0.40 to 1.16	0.71 to 1.45	0.32 to 1.26	0.60 to 1.54
*p*-Value	< 0.001	< 0.001	0.001	< 0.001

IL-1β				

β-Coefficient	0.71	0.92	0.70	0.93
95% CI	0.21 to 1.20	0.43 to 1.41	0.02 to 1.38	0.25 to 1.61
*p*-Value	0.006	< 0.001	0.043	0.009

TNFα				

β-Coefficient	0.78	0.53	0.75	0.51
95% CI	0.37 to 1.19	0.07 to 1.02	0.14 to 1.36	−0.17 to 1.20
*p*-Value	< 0.001	0.025	0.017	0.13

IFNγ				

β-Coefficient	0.64	0.58	0.62	0.55
95% CI	0.21 to 1.06	0.11 to 1.04	−0.01 to 1.26	−0.13 to 1.24
*p*-Value	0.004	0.015	0.05	0.11

Leptin				

β-Coefficient	0.04	0.31	0.04	0.31
95% CI	−0.09 to 0.17	0.18 to 0.43	−0.12 to 0.20	0.15 to 0.47
*p*-Value	0.57	< 0.001	0.61	< 0.001

CI, confidence interval. Outcomes and exposure variables were natural log transformed.

a Adjusted for mother age, SES, and TC.

**Table 3:** Multivariable adjusted analysis of associations of maternal U-As (μg/L in early and late pregnancy) and placental 8-oxoG (ACIA score) with placental effect markers (ACIA score).

	Independent variable						
	Unadjusted			Adjusted^a^		Adjusted^a^	
Dependent variable	U-As at GW8	U-As at GW30	8-oxoG	U-As at GW8	8-oxoG	U-As at GW30	8-oxoG
Leptin							

β-Coefficient	0.04	0.31	0.10	0.04	0.11	0.25	0.05
95% CI	−0.09 to 0.17	0.18 to 0.43	0.03 to 0.17	−0.22 to 0.13	0.03 to 0.18	0.07 to 0.44	−0.03 to 0.13
*p*-Value	0.57	< 0.001	0.005	0.61	0.008	0.008	0.19

IL-1β							

β-Coefficient	0.71	0.92	0.32	0.50	0.26	0.71	0.20
95% CI	0.21 to 1.20	0.43 to 1.41	0.06 to 0.59	−0.2 to 1.2	−0.05 to 0.57	−0.03 to 1.47	−0.11 to 0.52
*p*-Value	0.006	< 0.001	0.018	0.15	0.09	0.06	0.19

TNFα							

β-Coefficient	0.78	0.53	0.36	0.52	0.29	0.15	0.35
95% CI	0.37 to 1.19	0.07 to 1.02	0.12 to 0.58	−0.08 to 1.12	0.02 to 0.56	−0.56 to 0.85	0.05 to 0.64
*p*-Value	< 0.001	0.025	0.004	0.084	0,034	0.67	0.025

IFNγ							

β-Coefficient	0.64	0.58	0.38	0.37	0.31	0.19	0.34
95% CI	0.21 to 1.06	0.11 to 1.04	0.15 to 0.60	−0.26 to 1.0	0.04 to 0.60	−0.52 to 0.90	0.04 to 0.64
*p*-Value	0.004	0.015	0.002	0.23	0.029	0.59	0.029

CI, confidence interval. Outcomes and exposure variables were natural log transformed.

a Adjusted for mother age, SES, and TC.
